# SFMetrics: an analysis tool for scanning force microscopy images of biomolecules

**DOI:** 10.1186/s12859-015-0457-8

**Published:** 2015-01-28

**Authors:** Humberto Sánchez, Claire Wyman

**Affiliations:** Department of Genetics, Erasmus University Medical Center, Rotterdam, 3000CA The Netherlands; Department of Radiation Oncology, Erasmus University Medical Center, Rotterdam, 3000CA The Netherlands

## Abstract

**Background:**

Scanning force microscopy (SFM) allows direct, rapid and high-resolution visualization of single molecular complexes; irregular shapes and differences in sizes are immediately revealed by the scanning tip in three-dimensional images. However, high-throughput analysis of SFM data is limited by the lack of versatile software tools accessible to SFM users. Most existing SFM software tools are aimed at broad general use: from material-surface analysis to visualization of biomolecules.

**Results:**

We present SFMetrics as a metrology toolbox for SFM, specifically aimed at biomolecules like DNA and proteins, which features (a) semi-automatic high-throughput analysis of individual molecules; (b) ease of use working within MATLAB environment or as a stand-alone application; (c) compatibility with MultiMode (Bruker), NanoWizard (JPK instruments), Asylum (Asylum research), ASCII, and TIFF files, that can be adjusted with minor modifications to other formats.

**Conclusion:**

Assembled in a single user interface, SFMetrics serves as a semi-automatic analysis tool capable of measuring several geometrical properties (length, volume and angles) from DNA and protein complexes, but is also applicable to other samples with irregular shapes.

**Electronic supplementary material:**

The online version of this article (doi:10.1186/s12859-015-0457-8) contains supplementary material, which is available to authorized users.

## Background

Quantification of structural features from individual molecular complexes is used to explain their mechanism of action. Single-molecule imaging is a valuable tool in this sense because it reveals different stages of assembly and function. Functional assemblies of biomolecules are inherently flexible, variable and irregular. DNA and proteins are easily identified by SFM without staining procedures in conditions compatible with biochemical function (no vacuum, room temperature) [1]. Dozens of images with hundreds of molecules each can be routinely produced in one day. However there is no single software platform that allows semi-automatic and high-throughput data analysis. Usually data analysis requires the use of different, sometimes multiple, software programs none specifically designed for DNA and protein analysis.

Software analysis is generally provided as part of the microscope operating system software. For example, ‘NanoScope Analysis’ from Bruker and ‘JPKSPM Data processing’ from JPK Instrument software packages open their own generated images, allowing some basic filtering operations like flattening, but also other metrological operations like point to point length measurements, or volume analysis from selected particles. SPIP™ (Scanning Probe Image Processor- ImageMetrology) is a dedicated metrological tool for SFM that supports many formats but requires additional license payment. There are several open licensed software packages available for SFM. Image SXM was developed by S D Barret (http://www.ImageSXM.org.uk) in 1993 based on the image analysis software NIH Image (now ImageJ) [2]. Image SXM handles scanning microscope image files from more than 40 different instruments. It has many processing options and has been the standard SFM software analysis in many laboratories (including ours). However, it is limited to Mac OS system computers and some of the functions required for analysis of multiple particles in one image can be time-consuming. Unfortunately ImageJ software, compatible with many operating systems, did not evolve in the same manner and only a few plugins like ‘Open NV.java’ and ‘Open MI.java’ have been developed for opening SFM images. WSxM, developed by Nanotec in 2001 [3], is a Windows application for data acquisition and processing of scanning probe microscopes images. WSxM is compatible with most of the microscope manufacturers file formats and has common image processing operations, but it does not support high-throughput data analysis of individual complexes. Gwyddion [4] was developed in 2004 as a multiplatform for visualization and data analysis from scanning probe microscopes. Gwyddion runs in different operating systems and, among other multiple processing tools, allows statistical analysis of multiple particles detected with a variety of filters. However, selection of particles or measuring some individual features like contour length from irregular objects is not possible (see Table 1 for a comparison of these software tools).

**Table 1:** **A comparison of the features of current SFM image analysis software**

	**Image SXM**	**ImageJ**	**WSxM**	**Gwyddion**	**Nano-Scope**	**JPK-SPM**	**SFMetrics**
**Image format compatibility:**							
Bruker-Multimode	**Yes**	(Yes)*	**Yes**	**Yes**	**Yes**	**No**	**Yes**
JPK-NanoWizard	**Yes**	**No**	**Yes**	**Yes**	**No**	**Yes**	**Yes**
Asylum-MFP 3D	**Yes**	**No**	**Yes**	**Yes**	**No**	**No**	**Yes**
ASCII	**Yes**	(Yes)*	**Yes**	**Yes**	**No**	**No**	**Yes**
TJP(tiff/jpeg/png)	**Yes**	**Yes**	**Yes**	**Yes**	**No**	**No**	**Yes**
**Operating system:**							
OS X	**Yes**	**Yes**	**No**	**Yes**	**No**	**No**	**(Yes)** ^**&**^
Windows	**No**	**Yes**	**Yes**	**Yes**	**Yes**	**Yes**	**Yes**
Linux	**No**	**Yes**	**No**	**Yes**	**No**	**Yes**	**(Yes)** ^**&**^
**Irregular objects analysis:**							
Edge thresholding^#^	**No**	**No**	**No**	**No**	**No**	**No**	**Yes**
Skeleton-length	**No**	**No**	**No**	**No**	**No**	**No**	**Yes**
Freehand-length	**No**	**Yes**	**Yes**	**No**	**No**	**No**	**Yes**
**High-throughput analysis**	**Yes**	**No**	**No**	**Yes**	**No**	**No**	**Yes**

*ImageJ can make use of specific plugins to deal with these features.

^&^SFMetrics can be run from the scripts in any operating system with MATLAB installed.

^#^Although ‘edge detection’ is a common feature in many software packages it is usually not adjustable or cannot be used as a binary mask to extract features from the original image.

Here we present an open-licensed software for Windows using the stand-alone version (also Linux or OSX with MATLAB installed using the scripts) specifically aimed at the analysis of DNA and proteins. At the core of the software is a versatile edge detection algorithm that efficiently selects irregular objects from the background making volume and length determination straightforward. SFMetrics has been developed for efficient high-throughput analysis of individual particles with irregular shapes like biomolecules. Functionalities have been reduced to operations commonly applied to biomolecules (volume, contour length, height profile, width and angle measurement) for shortening the analysis time and for facilitating access for users unfamiliar with image processing techniques.

## Implementation

SFMetrics was developed in MATLAB (Release 2013b, The MathWorks, Inc., Natick, Massachusetts, United States) as a graphical user interface (GUI) for the geometrical analysis of DNA and protein molecules in SFM images. MATLAB scripts and the stand-alone version for Windows (64bit version) can be found here (http://cluster15.erasmusmc.nl/TIRF-SFM-scripts). If used under MATLAB, it requires MATLAB 2013b, or newer, Curve Fitting and Image Processing Toolboxes. If used as a stand-alone application, MATLAB Component Runtime (MCR) is required (available to download with the software package). It has a free license for non-commercial use.

### Image loading

SFMetrics can open image data files from NanoScope (v5.30v2), JPK (4.2.53), and Asylum Research microscopes by clicking ‘MultiMode’, ‘JPK’, or ‘Asylum’ button respectively. It can open NanoScope files using the MATLAB script ‘open_di.m’ (Jennifer R. Hampton, Hope College; details at http://nanoprobenetwork.org/) modified for extracting pixel resolution. JPK files have two images stored in a TIFF (Tag Image File Format) standard version 6.0 (http://partners.adobe.com/public/developer/en/tiff/TIFF6.pdf) that can be opened in MATLAB by using the ‘imread.m’ function (core MATLAB toolbox). The first image is a thumbnail version of the second containing the actual scan dimensions. JPK files are automatically calibrated by using the formula:

Result = offset + multiplier * value (Thomas Henze JPK instruments, personal communication).

Multiplier and the offset values are stored in the fields ‘Unknowntag(36)’ and ‘Unknowntag(37)’, as read by ‘imread.m’ respectively, if the image was saved with software versions previous to 4.2.53. If the image was saved with software version 4.2.53, the multiplier and the offset are in the fields ‘Unknowntag(37)’ and ‘Unknowntag(38)’ respectively. Asylum research files (Igor Pro file format- ibw) are opened using the MATLAB script ‘IBW.m’ (developed by Jakub Bialek and available at MATLAB central http://www.mathworks.nl/matlabcentral/). ASCII (American Standard Code for Information Interchange) files with pixel coordinates (xy) and intensity (z), including or not header lines, can be opened with the ‘ASCII’ button. TIFF, JPEG, and PNG files can be opened with the ‘TJP’ button that calls ‘imread.m’ MATLAB function. Example images files in the different formats can be found as Additional files: NanoScope (Additional file 1), JPK (Additional file 2), Asylum Research (Additional file 3), ASCII (Additional file 4), ASCII with header (Additional file 5), TIFF (Additional file 6), JPEG (Additional file 7), PNG (Additional file 8). After opening the image a smaller area is selected for the analysis.

### Basic operations

The selected area is loaded in the main panel so individual complexes can be measured. Each complex can be labelled with a specific alphanumeric identifier and, after cropping, it will appear in the adjacent window for volume and length analysis. Volume of the particle is calculated as follows: First, the cropped image is used to detect particles by performing a Sobel edge detection operation [5]. Two consecutive convolutions are done for the vertical and horizontal derivatives of the image and the results combined by using the square root of the sum of the squares. Then the operation is repeated with a user-defined threshold that determines the minimum height value to be included in the detection step. Detected regions are filled after opening and closing morphological operations. Regions connected to image borders are suppressed and the final binary mask is used for calculation on the unmodified image. Background height is calculated with the average pixel value excluding the detected particles.

The particle occupying the larger area in the image is surrounded by a red line and selected for calculations. The volume is calculated by adding the volume for each pixel, height value minus the average background as described, multiplied by pixel area. The line tool can measure the distance along a path defined by freehand tracing. The height (intensity) of the pixels along this traced line is plotted in a new window after moving the ‘Profile line’ slider (see below). Average height along the line and standard deviation are shown on top of the graph. Width can be estimated by measuring directly one part of the molecule with the line tool. However, if a more precise measurement is desired, multiple measurements along the traced line are obtained after clicking in the ‘Profile line’ slider. This tool defines the size of lines orthogonal to the freehand traced line. For each of these orthogonal lines the height profile is extracted and fitted to a Gaussian distribution. The STD of the fit at each point is used for estimating the full width at half maximum (FWHM, that equals ~2.3 times the STD) and plotted versus the length of the freehand trace line. Average FWHM along the line and its associated standard deviation are shown on top of the graph. Dimensions of height profile and width along molecules can identify the presence of different components of varying sizes, like proteins bound to DNA. Contour length of molecules can be estimated as described above using the line tool. However, because of the intrinsic flexibility of biomolecules, freehand definition of the contour-length can be difficult. In order to calculate the molecule contour-length in an unbiased manner we included a skeletonization tool. This tool produces the minimum length occupied by the complex by removing pixels on the boundaries without breaking it apart, and can be adjusted by manually changing the threshold intensity (height) and defining a minimum number of pixels to be taken into account in the calculations. Organization of irregular particles can be defined by describing the angle between specified segments based on user-defined points on the skeletonized molecule. All of these measurements can be exported in a Microsoft Excel spreadsheet file by a single click in the main panel. The Microsoft Excel spreadsheet file is created by using the MATLAB script ‘xlwrite.m’ (developed by Alec Zegher) without the necessity of having Microsoft Excel installed. Data for each complex is appended in different rows using the MATLAB script ‘xlsappend.m’ (Brett Schoelson, The MathWorks Inc). An isolated image of the complex under analysis, both as a two-dimensional and as a three-dimensional projection, can be saved in TIFF format by clicking ‘Save image’ button. For displaying purpose, image resolution is artificially increased by 10 fold using cubic interpolation of the four-by-four nearest-neighbour pixels. Color scale in the three-dimensional projection is compatible with red-green color perception deficiencies [6]. When finished with one area a new one can be defined and the analysis continued until the whole image is covered. Subsequently, a new image can be opened and the data will be stored in a new worksheet named with the image file-name in the same Microsoft Excel workbook.

### High-throughput analysis

For high-throughput analysis of particle-volume the program provides a tool (SFMedges) where edges of all particles in the whole image are detected as described above. SFMedges can be accessed after calling any of the image formats. The user can define the stringency of the detections by changing the threshold intensity. The volume of every particle in the image is calculated by adding the volume of each pixel in the detected area. Numerical data from all the particles (sum of pixel intensity, solidity or proportion of pixels actually occupied by the molecule in the polygonal region in which can be enclosed, pixel size in nanometers, ratio between minor/major axis of the ellipse enclosing the molecule, volume in cubic nanometers, eccentricity, length of the minor axis, and length of the major axis of the ellipse enclosing the molecule) are exported to an independent Microsoft Excel file so it can be sorted and analysed separately.

## Results and discussion

### Semi-automatic analysis of DNA and DNA-protein filaments

**Figure 1:**
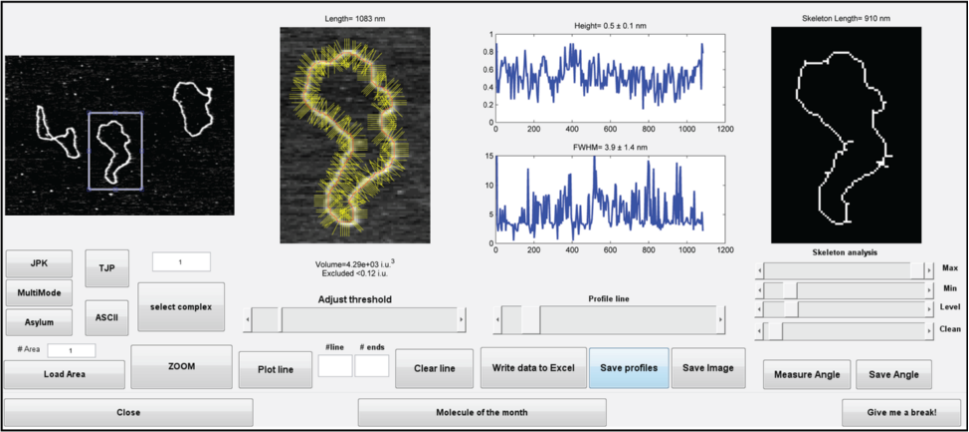
**SFMetrics main window screenshot for orientation and examples of different functions.** DNA molecules (pUC19) were used for obtaining the images analysed here. One DNA molecule was selected (white-blue square selection tool) for measuring the volume in the contiguous window where the pixels taken into account for volume calculation are surrounded by a red line after using ‘Plot line’ and defining ‘Line width’ (yellow lines). The graphs show the height and the FWHM, respectively, along the measured line. Skeleton length is shown in the right window.

**Figure 2:**
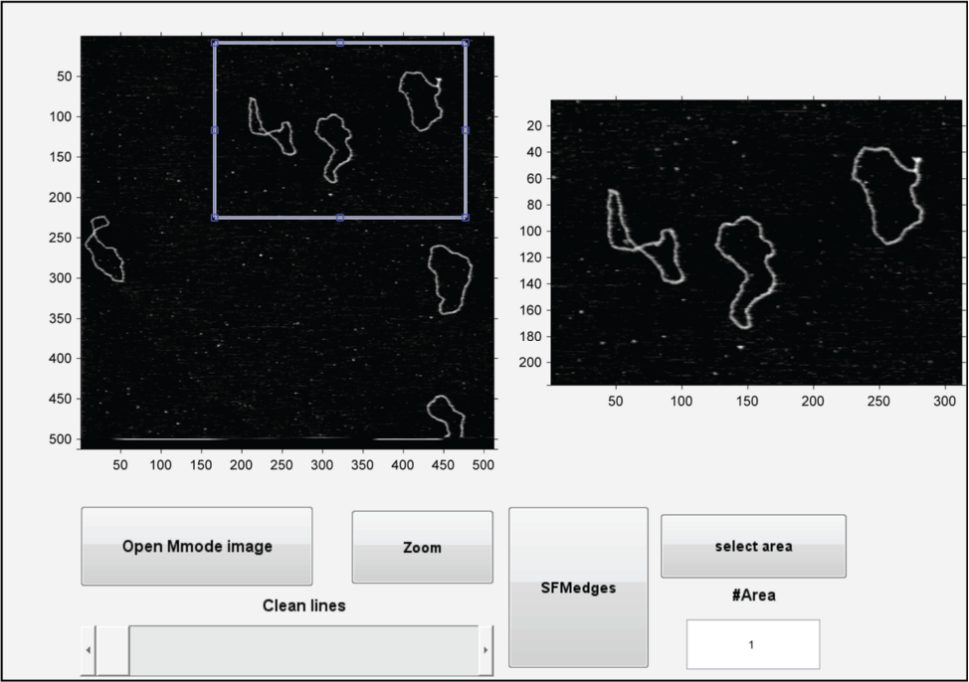
**SFMetrics image loading window, obtained by clicking image type (JPK, Multimode, Asylum, TJP, or ASCII) from main window (Figure** **1** **).** An area is chosen for analysis, shown as the white-blue square. The cropped area chosen for the analysis is displayed at the right (axis numbers refer to pixels).

**Figure 3:**
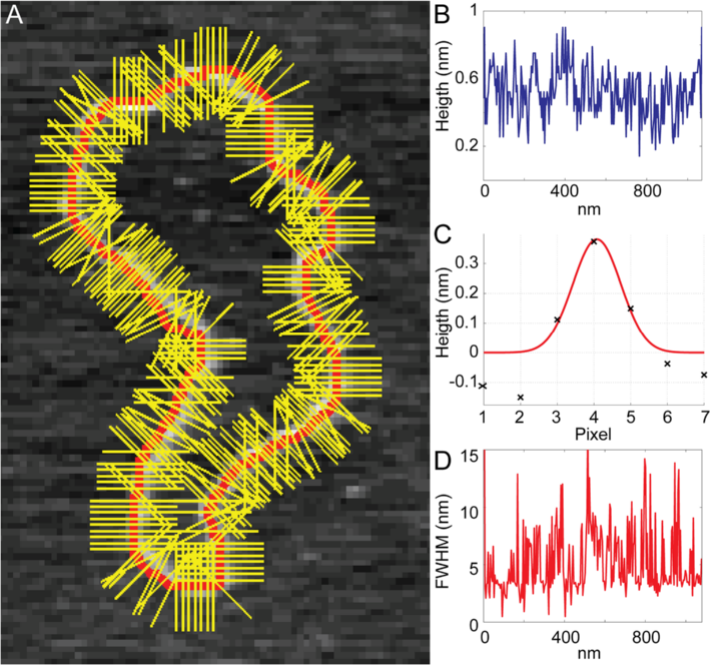
**Analysis of DNA dimensions with SFMetrics. A)** DNA contour-length of one DNA molecule (pUC19, center molecule in Figure 2 cropped area) has been traced by hand (red line) with the ‘Plot line’ tool. Orthogonal yellow lines to the freehand-traced line represent the cross-sections of the molecule used for extracting the height and for calculating the FWHM. **B)** DNA height along the freehand-traced. Maximum height from each cross-section (yellow lines in panel **A)** is plotted against length along the red line. **C)** Example analysis of one cross-section line from panel **A**. Height along the line (black crosses) is plotted against pixel position. Red line is the normal fitting to the data from where standard deviation is calculated and use for computing the FWHM. **D)** DNA width analysis along the freehand-traced. FWHM from each cross-section (yellow lines in panel **A**) is plotted against length along the red line.

**Figure 4:**
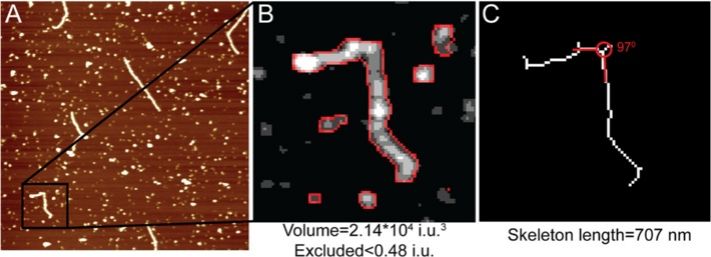
**Example analysing multiple features of DNA-protein complexes with SFMetrics. A)** SFM image of filaments formed by human RAD51 protein on double-stranded DNA. Image size: 2×2 μm, height is represented by color in the range of −2.5-2.5 nm. **B)** Volume analysis of the filament zoomed in panel **A**. The filament is surrounded by free protein complexes, not bound to DNA. However, these do not affect volume calculations performed only for the biggest object (the filament). i.u. = intensity units. **C)** Skeleton length tool is used for estimating the length of the filament. After appropriately adjusting the threshold slider only the filament axis is highlighted and background is discarded. Angle tool can be used to determine kink’s openness (in red).

**Figure 5:**
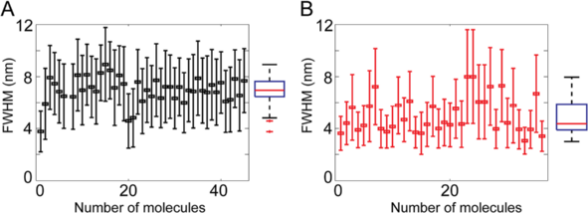
**Example of semi-automated analysis of biomolecules, effect of SFM-probe end radius of curvature on DNA width. A)** Measurements done with AppNano ACT-W probe (nominal radius of curvature 5 nm). Average FWHM from at least 200 cross-sections and STD (error bars) is plotted for each of the 45 DNA molecules (pUC19) analysed. Boxplot summarizing the data is depicted to the right side of the graph. Red line, median (6.9 nm); the edges of the blue box are the 25th (6.4 nm) and 75th (7.6 nm) percentiles, the whiskers extend to the most extreme data points not considered outliers (red crosses). **B)** Measurements done with Nanosensors SSS-NCH probe (nominal radius of curvature 2 nm) as in **A** from 36 DNA molecules (pUC19) analysed. Boxplot data: median = 4.4 nm; 25th percentile = 3.9 nm; 75th percentile = 5.9 nm.

SFMetrics function was tested using SFM image data of biomolecules typically generated in our laboratory. DNA fragments of 2 kbp were incubated with RAD51 protein, a strand exchange protein that binds DNA and forms stable defined nucleoprotein filaments in the conditions used [7]. Circular dsDNA (pUC19 plasmid), or the nucleoprotein filaments were deposited on freshly cleaved mica, rinsed with water and dried with filtered air [1]. Molecules were imaged in air using a Nanoscope III or IV (Bruker) operating in Tapping Mode™. SFM images were flattened to remove background slope by subtracting a 1st order plane fit with ‘NanoScope Analysis’ software (Bruker) and saved in the original format before opening in SFMetrics. Figure 1 shows a screen-shot of the SFMetrics starting window. After selecting the type of image (JPK, Multimode, Asylum, TIFF/JPEG/PNG, or ASCII) a new window for selecting specific parts of the image is opened (Figure 2). This is useful for large images filled with many objects to analyse. The selected area can be called by typing a number previously assigned by the user in the main window. Specific DNA (Figures 1 and 3) or DNA-protein complexes (Figure 4) are tagged with a number and selected with a square selection tool and the cropped region is displayed in the adjacent window. Volume of the object, in this case DNA or a RAD51 nucleoprotein filament, is calculated after detecting the edges (red contour-line in Figure 4B) of the complex using the sliding threshold tool. The ‘Plot line’ tool allows measurements of distances along a path defined by freehand tracing (red line in Figure 3A) and can be used for determining protein positions on DNA, for example. Height (Figure 3B) and width (Figure 3D) profiles from the line can be acquired by defining the desired width of a line perpendicular to the user-defined path (yellow line in Figure 3A). In order to illustrate this feature, we measured length, width and height of circular DNA molecules using two probes with different radius of curvature. Forty-five (from 15 images) and thirty-six DNA molecules (from 13 images) were measured with AppNano ACT-W probe (nominal radius of curvature 5 nm) and Nanosensors SSS-NCH probe (nominal radius of curvature 2 nm), respectively and hereafter probe A and probe B. Length measured by freehand tracing was 1000 ± 64 (STD) nm (probe A) and 991 ± 46 (STD) nm (probe B). These measurements were significantly (p < 0.05) longer than the skeleton length: 835 ± 64 (STD) nm (probe A) and 847 ± 68 (STD) nm (probe B) but similar between the probes. DNA molecules were 2686 bp in length corresponding to ~892 nm of B-form dsDNA. The different length measured accounted for an overestimation of about 10% of the expected length due to pixel oversampling and user inaccuracy when freehand tracing that did not happen in skeleton length determination. Radius of curvature of the probe, however, is expected to affect significantly the width of the scanned molecules. We measured the FWHM of the DNA molecules in every point along the freehand-traced line (at least 200 data points per DNA molecule) as described above. Figure 5 shows the results obtained with each probe. Probe A produced DNA images with a median FWHM of 6.9 nm (with 6.4 and 7.6 nm as 25th and 75th percentiles respectively), while probe B produced DNA images with a median FWHM of 4.4 nm (with 3.9 and 5.9 nm as 25th and 75th percentiles respectively), significantly thinner (p < 0.05). The average height of the DNA was 0.5 ± 0.08 (STD) nm and 0.3 ± 0.1 (STD), for probe A and B respectively.

The skeleton tool can be used to provide an estimation of the length occupied by the minimum number of pixels representing the object selected. In the case of nucleoprotein filaments presented in Figure 4, the skeleton is the axis of the DNA (Figure 4C). On the skeleton axis, the angle of kinks between different parts of the molecule can be measured with the angle tool (Figure 4C). All the different measurements associated with the selected object are saved in an Excel file with ‘Write data to Excel’ button. A new complex in the same image can be then be analysed and processed iterating as many times as desired. Measurements from the same image will be appended in the same Microsoft Excel file.

### High-throughput protein complex analysis

**Figure 6:**
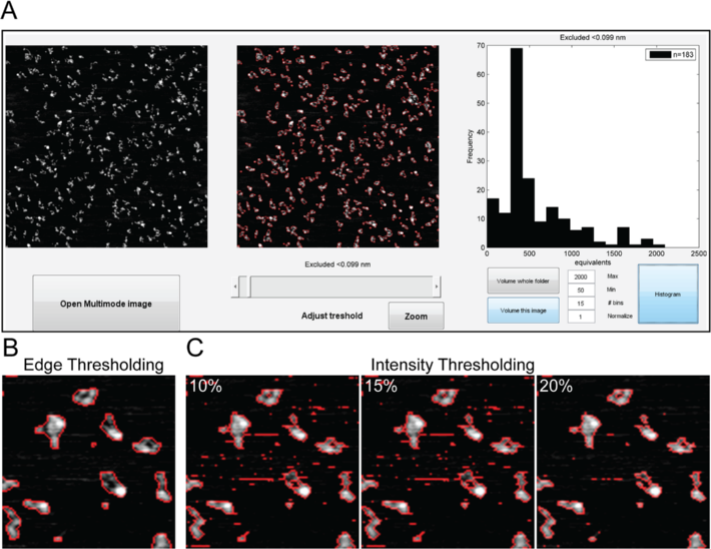
**Example of high-throughput analysis of protein complexes. A)** Screenshot of SFMedges (obtained by clicking on SFMedges button shown in Figure 2) showing the analysis of RAD50-MRE11 complexes deposited on mica. Selected complexes for the analysis are delimited by a red line after the user has determined the selection stringency with ‘Adjust threshold’. This results in a histogram showing the volume distribution of the selected complexes, displayed at the right. The data has been normalized to kiloDaltons (kDa) using the volume of E.coli RNA polymerase (450 kD) determined separately as a standard. The histogram here has been limited to display particles larger than 50 kDa and smaller than 2000 kDa, and the number of bins set at 15. **B)** Zoom of the image in panel **A** after detecting the edges of the complexes (zoom size: 390x390 nm). **C)** Comparison with other threshold methods. Same zoomed area as in panel **B** analysed by direct intensity thresholding discarding pixels with values below 10%, 15%, and 20% of the maximum image intensity as conventionally applied.

Many protein complexes have irregular shapes making quantification of individual features challenging. The protein complex made of MRE11 and RAD50 (MR) has a characteristic architecture defined from SFM images, some features of which determine its function in binding and tethering DNA molecules for repair of double-strand breaks [8]. Stoichiometry of MR has been defined by a combination of volume analysis and identification of long coiled-coils protruding from RAD50 as seen by SFM. In order to test the high-throughput potential of the software, we used images of the MR complex. The purified protein complex was deposited onto freshly cleavage mica, (as described in ref [9]). Molecules were imaged as described in the previous section. Figure 6A shows a screen-shot of the module for high-throughput analysis, called by pressing ‘SFMedges’ in the panel after opening the image (Figure 2). In this case, the edge detection algorithm works on the whole image detecting all particles (middle panel in Figure 6A and zoom in Figure 6B). The threshold stringency for selecting the particles is defined by the user ignoring edges below the threshold. Because the edge detection algorithm operates on the differences between neighbouring pixels using an image gradient, rather than on the pixel values directly, molecule detection is less sensitive to noise compared to conventional intensity thresholding (Figure 6C). Volume data for the detected particles is stored in a file and its distribution displayed in a user-adjustable histogram. This histogram can reveal if a protein preparation is mono-disperse or not, for example. However, due to tip-sample convolution effect, absolute volume determination by SFM requires calibration with known standards. If several images have been collected in automatic mode and stored in the same folder, volume analysis can be performed in batch after selecting the ‘Edge factor’ with one of the images, and by clicking in the ‘Volume whole folder’ button. One single image can be analysed by clicking ‘Volume this image’. Volume distribution can be used for identifying different populations of molecules in the scanned area. Maximum and minimum volume to be analysed, together with the number of bins in the histogram, can be adjusted by typing the desired numbers.

### Comparison with other tools

Proper calibration of SFM images is of the utmost importance to recover accurate data from the recorded images. Each manufacture uses a different file format for storing the pixel size and height. Although pixel size calibration is straightforward after knowing the scan size, pixel height calibration is not so obvious. Differences in height are represented in the images by differences in pixel intensity stored usually as 16 bit values, but also as 8 bit or 32 bit, and as integer or real numbers. The information that makes the file readable is attached in the file as text or binary data. These differences make it difficult to open SFM images in software other than that provided by the manufacturer. Thus any software for analysis of SFM images has to be able to extract image calibration information for the data files.

The software presented here shares functionalities with Image SXM, an ImageJ derivative that runs as freeware in Mac OS operation systems. A detailed protocol for measuring protein volume from SFM images using Image SXM has been published [10]. SFMetrics can perform similar calculations in a straightforward manner in a Windows platform using the stand-alone version (also Linux or OSX with MATLAB installed using the scripts) with the added benefit of integrating a range of metrology tools. Measurement of distances point to point is a common characteristic in the available programs, however a tool to trace paths freehand offers more capabilities. High-throughput analysis of the data, such as position along a path and height profiles along a path, with the existing programs is challenging and time consuming. The edge detection tool, easily adjustable by the user, makes SFMetrics a powerful tool for fast and reliable analysis of biomolecules in SFM images. It accurately defines the area to be analysed occupied by very irregular objects typical of complex biomolecular assemblies. Analysis of SFM data has typically employed custom written routines developed in each laboratory, using different procedures for determining the volume or other dimensions of individual particles [9-15].

Our software is intended for a broad spectrum of potential users, although it has been developed specifically for the analysis of SFM images of biomolecules, it could also be applied to any objects or image features that are irregular and variable. It does not require programming skills but can be adjusted for different purposes with minimum modifications in MATLAB, a common software environment used in image and matrix analysis.

## Conclusions

SFMetrics is a semi-automatic analysis tool capable of measuring several geometrical properties (length, volume and angles) from DNA and protein complexes. It is assembled in a single user interface provided as a MATLAB script and as a stand-alone application running in Windows. We have developed a versatile tool to analyse irregular objects applying user-adjusted thresholds. Multiple features of the same complex can be linked in a tabulated form, so that the user does not have to keep track of all the measurements and link them for analysis manually. SFMetrics enables semi-automatic and high-throughput analysis of SFM data, specifically aimed to DNA and proteins, that can be easily adapted to other samples.

## Availability and requirements

**Project name:** SFMetrics

**Project home page:**http://cluster15.erasmusmc.nl/TIRF-SFM-scripts

**Operating system:** Windows (64bit version) for the stand-alone version

**Programming environment:** MATLAB R2013b (64bit version)

**Requirements:** If used under MATLAB: MATLAB 2013b or newer, Curve Fitting and Image Processing Toolboxes are required. If used as a stand-alone application, MATLAB Component Runtime (MCR) is required (available to download with the software package).

**License:** Free for non-commercial use.

## Additional files

Additional file 1:
**SFM example image of DNA plasmid in NanoScope format.**
Additional file 2:
**SFM example image of DNA plasmid in JPK format.**
Additional file 3:
**SFM example image of nuclear membrane in Asylum Research format.** Image courtesy of S Yoshimura and S. Otsuka (Kyoto University). Additional file 4:
**SFM example image of DNA plasmid in ASCII format.**
Additional file 5:
**SFM example image of DNA plasmid in ASCII with header format.**
Additional file 6:
**SFM example image of DNA plasmid in TIFF format.**
Additional file 7:
**SFM example image of DNA plasmid in JPEG format.**
Additional file 8:
**SFM example image of DNA plasmid in PNG format.**

## Abbreviations

TIFF: Tag image file format
SFM: Scanning force microscopy
FWHM: Full width at half maximum
STD: Standard deviation
Kbp: Kilobase pair
