# Supplementary figures and images for: SFMetrics: an analysis tool for scanning force microscopy images of biomolecules
Source: BMC Bioinformatics. 2015 Jan 28;16(1):27. doi: 10.1186/s12859-015-0457-8 (PMC4314933; doi:10.1186/s12859-015-0457-8)

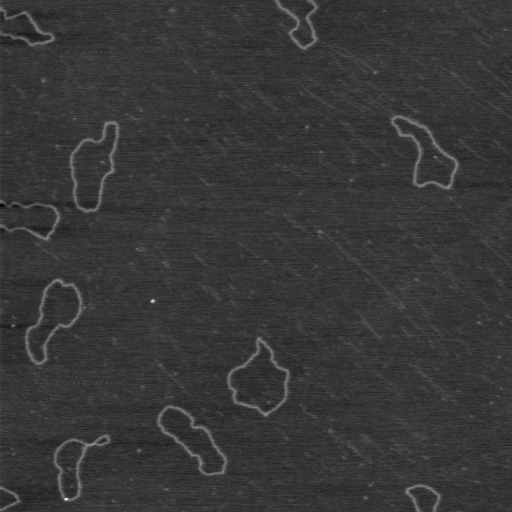

Supplement: Additional file 6: — SFM example image of DNA plasmid in TIFF format. [file 12859_2015_457_MOESM6_ESM.tiff]

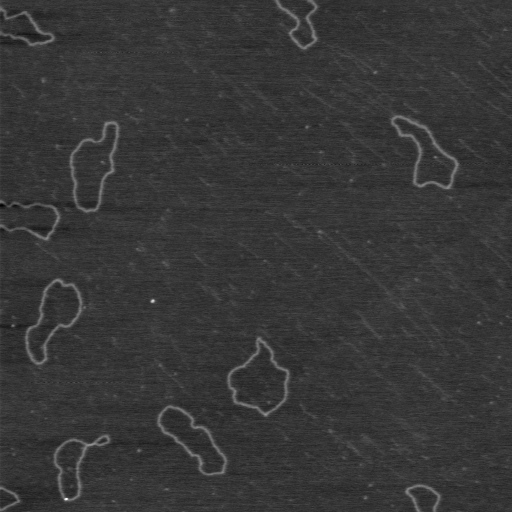

Supplement: Additional file 7: — SFM example image of DNA plasmid in JPEG format. [file 12859_2015_457_MOESM7_ESM.jpeg]

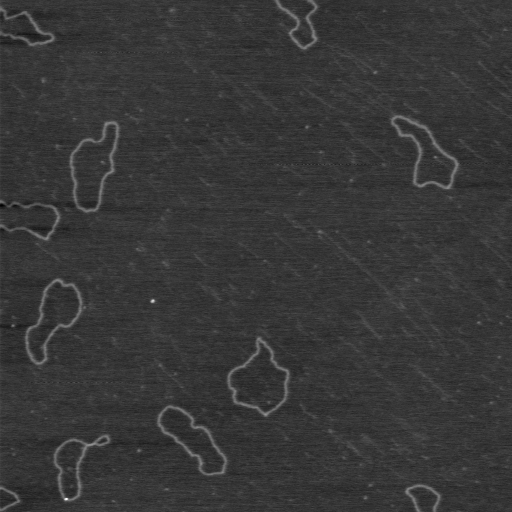

Supplement: Additional file 8: — SFM example image of DNA plasmid in PNG format. [file 12859_2015_457_MOESM8_ESM.png]
